# Nanoscale imaging of pT217‐tau in aged rhesus macaque entorhinal and dorsolateral prefrontal cortex: Evidence of interneuronal trafficking and early‐stage neurodegeneration

**DOI:** 10.1002/alz.089418

**Published:** 2025-01-03

**Authors:** Dibyadeep Datta

**Affiliations:** ^1^ Yale University, New Haven, CT USA

## Abstract

**Background:**

Advances in Alzheimer’s disease (AD) have revealed a novel fluid biomarker, tau phosphorylated at T217 (pT217‐tau), in CSF and plasma, that predicts AD prior to cognitive deficits. Understanding the role of pT217‐tau is important in assessing efficacy of novel treatments aimed at early‐stage disease. However, it is unknown why pT217‐tau is effective in predicting brain pathology, as little is known about early, soluble pT217‐tau brain expression. These questions are difficult to address in humans, as soluble p‐tau is rapidly dephosphorylated postmortem, and PET scans detect late‐stage, fibrillated tau. However, the etiology of pT217‐tau in aging brains can be probed in rhesus macaques, where perfusion fixation allows capture of phosphorylated proteins in their native state. Aging macaques naturally develop tau pathology with the same qualitative pattern and sequence as humans, including initial cortical pathology in layer II of the entorhinal cortex (ERC) evident early in aging, and later in layer III of the dorsolateral prefrontal cortex (dlPFC).

**Method:**

We utilized multi‐label immunofluorescence and immunoelectron‐microscopy to examine the subcellular localization of early‐stage pT217‐tau in ERC and dlPFC of aged macaques with naturally occurring tau pathology and assayed pT217‐tau levels in plasma.

**Result:**

Our results show that pT217‐tau labeling is primarily observed in postsynaptic compartments, accumulating in: 1) dendritic spines on the calcium‐storing smooth endoplasmic reticulum spine apparatus near asymmetric glutamatergic‐like synapses, and 2) in dendritic shafts, where it aggregated on microtubules, often “trapping” endosomes associated with Aβ42. The dendrites expressing pT217‐tau were associated with autophagic vacuoles and dysmorphic mitochondria, indicative of early neurite degeneration. We observed trans‐synaptic pT217‐tau trafficking between neurons within omega‐shaped bodies and endosomes, specifically near excitatory, but not inhibitory synapses. We also examined pT217‐tau in blood plasma in macaques across age‐span and observed a statistically significant age‐related increase in pT217‐tau.

**Conclusion:**

These data provide the first direct evidence of pT217‐tau trafficking between neurons near synapses to “seed” tau pathology in higher brain circuits, interfacing with the extracellular space to become accessible to CSF and blood. The expression of pT217‐tau in dendrites with early signs of degeneration may help to explain why this tau species can herald future disease.

